# Draft Genome Sequence of the Novel Black-Pigmented *Planococcus* sp. Strain CAU13

**DOI:** 10.1128/genomeA.01160-14

**Published:** 2014-12-24

**Authors:** Cameron A. Unverferth, Ian C. Santisteban, Aaron T. Setterdahl

**Affiliations:** Indiana University Southeast, New Albany, Indiana, USA

## Abstract

Presented here is the whole-genome sequence of a previously uncharacterized species of the genus *Planococcus*. A 16S sequence analysis shows that this bacterium exhibits 98% sequence identity to the closest relative of *Planococcus kocurii*. Whereas most species of *Planococcus* produce yellow to orange pigments, the species described here produces black pigmentation.

## GENOME ANNOUNCEMENT

Environmental soil and sand samples obtained during a field geology expedition to the Hashemite Kingdom of Jordan (29.31ʹN 35.25ʹE) were analyzed to characterize desert microbial communities. An investigation of the microbial community revealed the presence of black pigmentation in a previously uncharacterized bacterium. A 16S sequence comparison of the novel bacterium by BLAST demonstrated it to be closely related to the *Planococcus* or *Planomicrobium* genus (1, 2). The closest relatives in the *Planococcus* genus as determined by 16S sequence analysis, with 98% identity, are *P. kocurii* (GenBank accession no. X62173.1) (3), *P. citreus* (GenBank accession no. X62172.1) (3), and *P. maritimus* (GenBank accession no. AF500007.1) (4).

Genomic DNA of *Planococcus* sp. strain CAU13 was purified using the PureLink genomic DNA purification kit (Invitrogen), according to the protocol for Gram-positive bacterial purification. Purified genomic DNA was used to construct a library and 76-bp paired-end sequencing on an Illumina HiSeq from htSEQ in Seattle, WA, USA. *De novo* assembly with 24× coverage gave a genome size of 3,310,887 bp using 355 contigs >200 bp. Analysis by the RAST server and SEED revealed 3,355 coding sequences (5, 6). Notable subsystem features include 37 genes involved in resistance to antibiotics and toxins.

### Nucleotide sequence accession numbers.

This whole-genome shotgun project has been deposited at DDBJ/EMBL/GenBank under the accession no. JRGN00000000. The version described in this paper is version JRGN01000000.
